# The effect of distance between holes on the structural stability of subchondral bone in microfracture surgery: a finite element model study

**DOI:** 10.1186/s12891-020-03467-z

**Published:** 2020-08-18

**Authors:** Xiang Yun Yin, Do Young Park, Young Jick Kim, Hye Jung Ahn, Seung-Hyun Yoo, Byoung-Hyun Min

**Affiliations:** 1 Department of Orthopedic Surgery, School of Medicine, Ajou University, Suwon, Republic of Korea; 2 Cell Therapy Center, Ajou University Medical Center, Suwon, Republic of Korea; 3 Department of Molecular Science and Technology, Ajou University, Suwon, Republic of Korea; 4 Department of Mechanical Engineering, college of Engineering, Ajoy University, Suwon, Republic of Korea

## Abstract

**Background:**

Microfracture is a surgical technique that involves creating multiple holes of 3–4 mm depth in the subchondral bone to recruit stem cells in the bone marrow to the lesion, inducing fibrocartilage repair and knee cartilage regeneration. Recently, it has been reported that increasing the exposed area of the lower cartilaginous bone (drilling a lot of holes) increases the outflow of stem cells, which is expected to affect the physical properties of the subchondral bone when the exposed area is large. The purpose of this study was to analyse the effect of the distance between the holes in the microfracture procedure on the structural stability of the osteochondral bone using a finite element method.

**Methods:**

In this study, lateral aspects of the femoral knee, which were removed during total knee arthroplasty were photographed using microtomography. The model was implemented using a solitary walks program, which is a three-dimensional simplified geometric representation based on the basic microtomography data. A microfracture model was created by drilling 4 mm-deep holes at 1, 1.5, 2, 2.5, 3, 4, and 5 mm intervals in a simplified three-dimensional (3D) geometric femoral model. The structural stability of these models was analysed with the ABAQUS program. We compared the finite element model (FEM) based on the microtomography image and the simplified geometric finite element model.

**Results:**

Von Mises stress of the subchondral bone plate barely increased, even when the distance between holes was set to 1 mm. Altering the distance between the holes had little impact on the structural stability of the subchondral bone plate. Safety factors were all below 1.

**Conclusions:**

Although we did not confirm an optimal distance between holes, this study does provide reference data and an epidemiological basis for determining the optimal distance between the holes used in the microfracture procedure.

## Background

Knee cartilage damage may be caused by several factors, from acute trauma such as sports injuries to chronic conditions such as degenerative arthritis [1, 2]. Various clinical treatments are currently available, such as microfracture, autologous chondrocyte implantation, and mosaicplasty [3]. Despite recent advances in treatment, microfracture remains the standard surgical technique for small chondral defects due to its cost effectiveness and simplicity [4, 5]. The microfracture technique involves stimulation of bone marrow beneath the subchondral bone. After removing unstable cartilage from the lesion, multiple holes of 3–4 mm depth are made about 3–4 mm apart using an awl to recruit stem cells in the bone marrow to the lesion, which induces fibrocartilage repair [6, 7].

Several surgical factors have been found to affect cartilage repair after microfracture. One report suggested that the volume of mesenchymal stem cells (MSCs) migrating to the defect through the micro-perforations affect the outcome of cartilage regeneration. Another study stated that the MSC count in the lesion area and treatment outcomes vary in relation to the size and number of holes made [8]. Hoemann et al. investigated the effects of hole depth of microfractures in promoting cartilage repair, and found that a hole depth of 4 mm was effective for cartilage repair [7]. We have previously found that increasing the surface area of bone marrow stimulation by adjusting the size and quantity of holes in the subchondral bone plate led to the greatest count of MSC drained from microfracture holes. In other words, having more exposure areas is beneficial for draining MSCs [8]. Draining too many holes, however, may damage the mechanical stability of the subchondral bone, as the knee is subject loading by the body’s weight, and this may ultimately hinder cartilage repair. Unfortunately, there is not much research data on the mechanics regarding bone marrow stimulation procedures. Furthermore, although surgeons are recommended to maintain a 3–4 mm distance between holes during microfracture, this recommendation is purely empirical and is not supported by mechanical research data. Biomechanical studies investigating the effects of the number, size, depth and interval of holes during microfracture on the structural stability of the subchondral bone are required.

In efforts to identify the mechanical stability after microfracture, we hypothesised that the distance between holes in microfracture would correlate with the structural stability of the subchondral bone. We assessed the structural stability of the subchondral bone by establishing a three-dimensional geometric FEM of the joint using a micro-CT-based modelling technique and analysing the changes of stress and displacement in relation to the distance between micro-perforations via a finite element analysis.

## Methods

### 3D image generation and structural analysis of osteochondral tissue

**Fig. 1:**
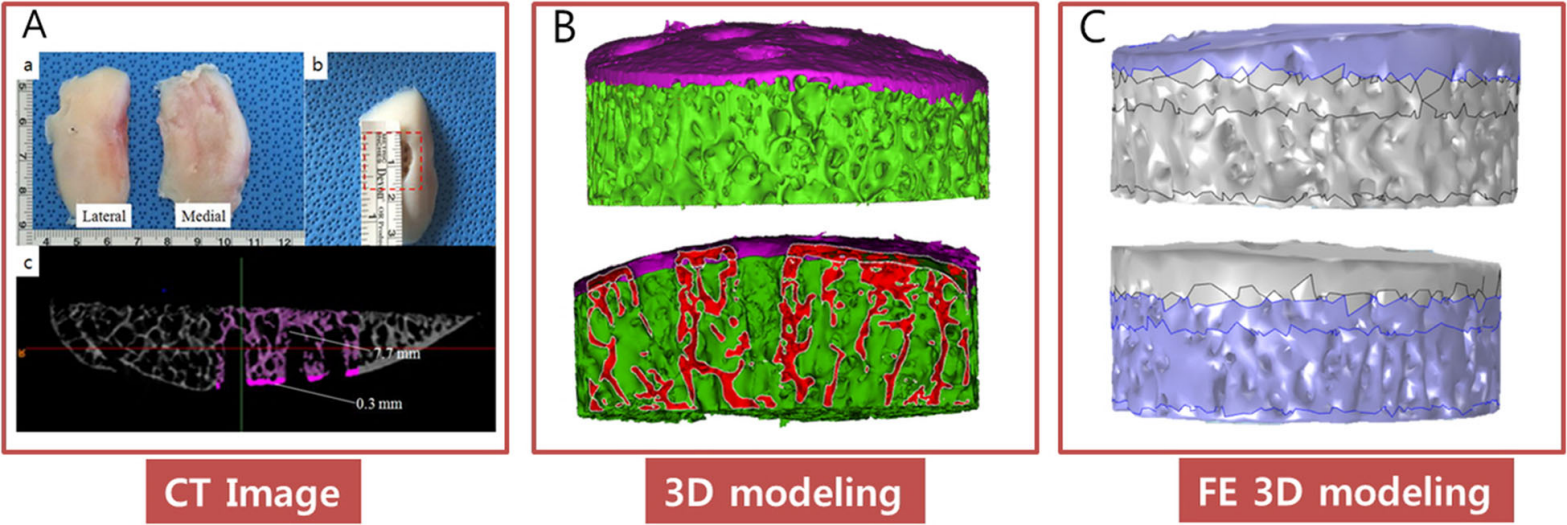
Preparation of microfracture samples. **a** a. Lateral and medial aspects of the femoral osteochondral block, b. Femoral condylar block with chondral defect created and treated with microfracture, c. Micro-CT images; **b** 3D modelling; **c** FE modelling. CT, computed tomography; FE, finite element

This study was approved by our institutional review board (AJIRB-BMR-SMP-14-125). The study was conducted using donated lateral aspects of femoral knee removed from patients who underwent total knee arthroplasty (TKA) due to osteoarthritis or knee injury (Fig. 1a). The donated lateral aspects of femoral knees were photographed using a Micro-CT Scanner (SkyScan1076, Bruker Miro-CT NV, Aarselaar, Belgium) at a voltage of 40 kVp, current of 200 Ma and 200 ms integration time, using an 0.5-mm-thick aluminium filter at a resolution of 18 um. To establish a model that accurately reflects the actual state for the finite element analysis, the CT images were 3D modelled using the Mimics software (Materialise’s interactive medical image control system; Leuven, Belgium). To compare microfracture, the images of the cartilage, subchondral bone and trabecular bone layers must be distinguishable [9]. However, we removed the cartilage layer because the purpose of this study was to examine the maximum compression stress that is placed on areas near the hole after microfracture surgery. Images of the trabecular bone and subchondral bone layer were independently constructed to compare microfracture procedures. As shown in Fig. 1, in order to compare the stress between the micro-fracture hole and the hole, we use the Mimics program 16.0 software to construct the subchondral bone and trabecular bone layer independently, but the subchondral bone could not be separated from the computed tomography (CT) image because it is under the cartilage. The bone was divided into 0.3-mm sections based on the CT data of the TKA patient. However, the three-dimensional reconstruction of CT images poses two challenging problems for its application in finite element analysis. Firstly, it is difficult to identify two different levels from the CT data, namely the subchondral and trabecular separation areas, and secondly, there is a degree of discontinuity between the subchondral bone and trabecular layers, which causes fatal errors in structural analysis. In other words, it was difficult to distinguish between the subchondral bone and trabecular bone as shown in Fig. 1c. Therefore, we used a simplified 3D model that resolves such a problem for this study.

### Finite element method

**Fig. 2:**
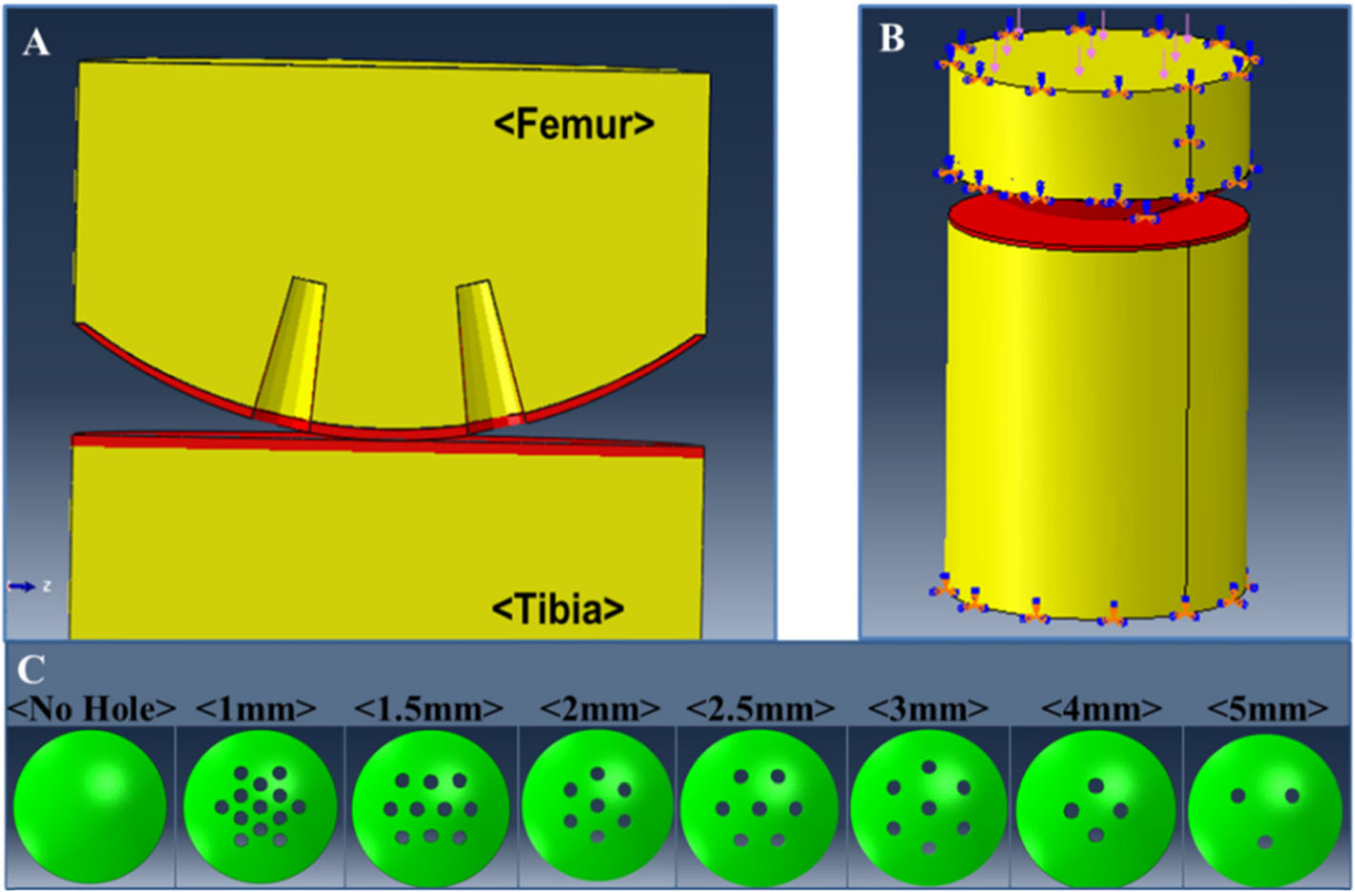
Simplified imaginary model. **a** Simplified imaginary knee articular cartilage model; **b** Boundary conditions; **c** Number of holes in each group

Figure 2 shows the schematic diagram of the 3D knee joint model. A finite element modelling (i.e., ABAQUS 6.11.1, SIMULIA, Providence, RI, USA) method is used to assess maximum compression stress on a structure produced by microfracture. The 3D model used for the finite element analysis comprised two domains: subchondral bone and trabecular bone. Because this study aimed to examine maximum compression stress in relation to the distance between holes made in the microfracture procedure, we assumed that there was no subchondral bone tissue or trabecular bone damage caused when making micro-perforations using an awl. Based on data obtained from analysis of micro-CT images of actual human samples, a simplified 3D imaginary model was established by setting the thicknesses of the subchondral bone and trabecular bone to 0.3 mm and 7.7 mm, respectively (Fig. 1c). In addition, the distance between the holes in the imaginary models was set to 1 mm, 1.5 mm, 2 mm, 2.5 mm, 3 mm, 4 mm and 5 mm, with a depth of 4 mm as if actual microfracture was performed (Fig. 2c). Different materials were used in this simulation: isotropic linear elastic with the elastic modulus of 17 GPa and 700 MPa for subchondral bone and trabecular layers, respectively. Poisson’s ratios were 0.3 and 0.25 for both subchondral and trabecular bones, respectively [10] (Table 1). To establish a biomechanical environment in the light-to-femoral joint, we assumed that an equal force is placed on the medial and lateral aspects of the knee. Based on this assumption, we applied a force in the direction of the tibia on the upper surface of the femur while placing a fixed constraint in all directions on the lower surface of the tibia to ensure that the underside remained fixed. In addition, we assumed that all contact surfaces were frictionless. The size of the load was set to 147 N [15], which is equal to the load inflicted when humans stand on two feet. In this study, the ABAQUS software was used for the finite element analysis. Based on the von Mises stress that was obtained from the 3D element analysis, the peak von Mises stress distribution and displacement were analysed for each model, as shown in Fig. 2c.

**Table 1:** Material Properties

	Young’s modulus	Poisson’s ratio	References
Subchondral bone plate femur and tibi	17 [GPa]	0.3	[11–13]
Trabecular bone of femur and tibia	700 [MPa]	0.3	[14]

## Results

### Analysis of stress distribution in relation to distance between holes in a simplified imaginary model

**Fig. 3:**
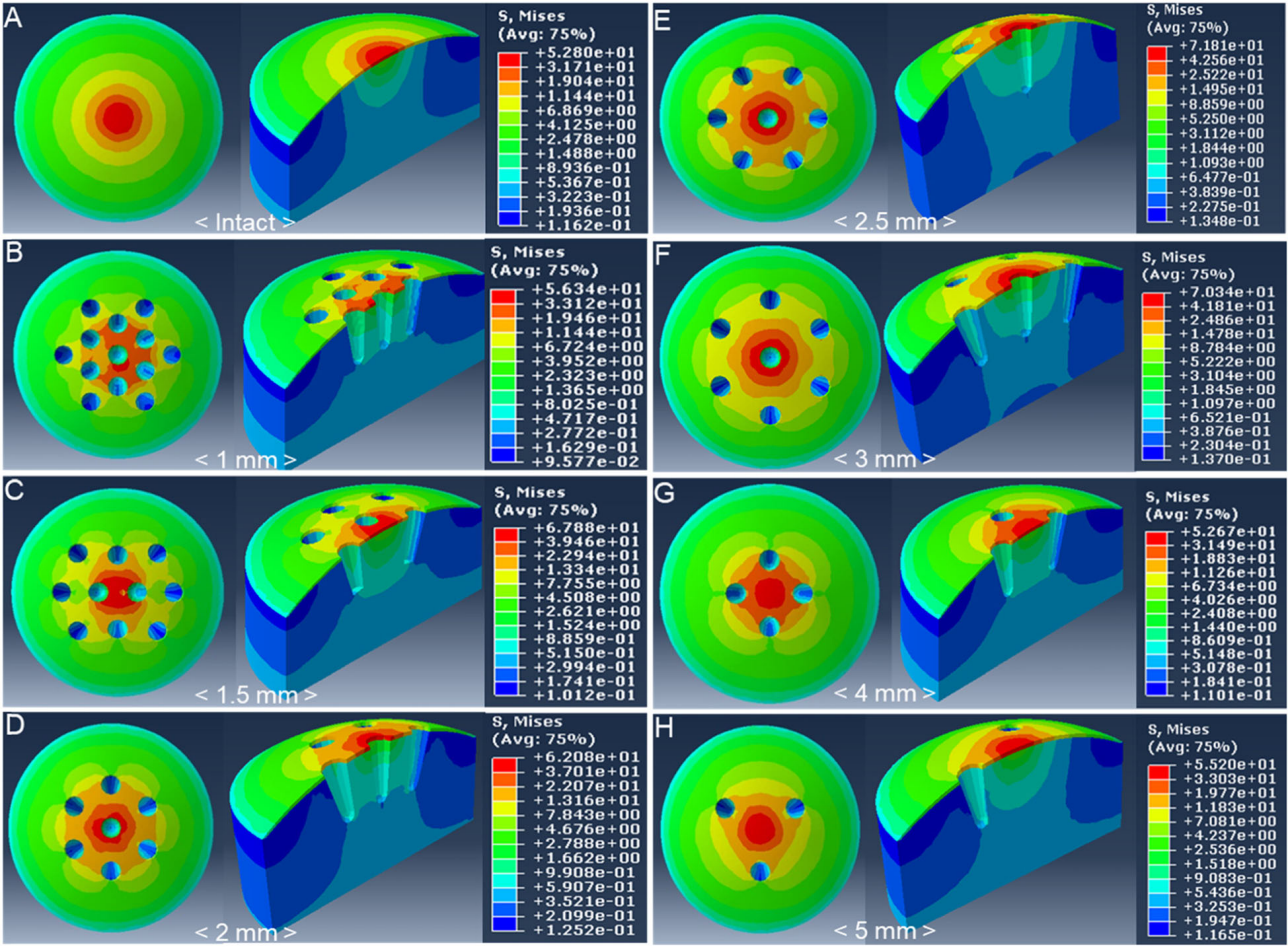
Von Mises Stress distribution in the simplified 3D imaginary model. **a** Model of intact defect without holes; **b** ~ **h** Microfracture models with 1 mm, 1.5 mm, 2 mm, 2.5 mm, 3 mm, 4 mm, and 5 mm distance between holes

The results were interpreted under the premise that the FEM is homogenous, isotropic and linear elastic. In each imaginary model, the distribution of von Mises stress was analysed with finite element analysis to examine the structural stability of the subchondral bone plate in relation to the distance between the holes. The intact group showed stress inflicted concentrically within a small range (Fig. 3a). Similarly, stress was inflicted concentrically in the other groups with holes, but the ranges were larger than that shown in the intact group. Further, stress was inflicted in a greater range in groups with holes on the contact area compared to those without holes on the contact area (Fig. 3 b-h).

### Analysis of peak Von Mises stress in relation to distance between holes in simplified imaginary model

**Fig. 4:**
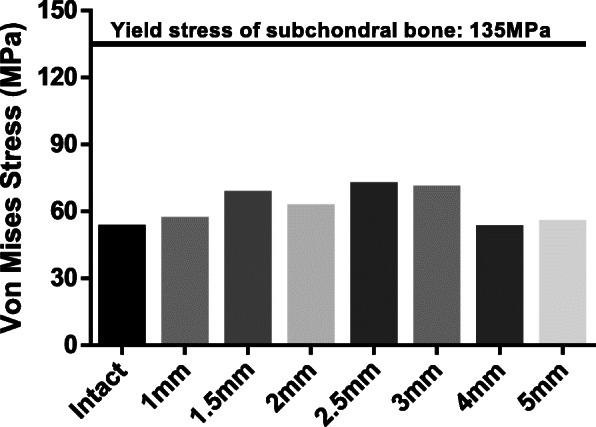
Peak von Mises stress in the XY cross-section in the 3D imaginary model

The peak von Mises stress on the subchondral bone plate was 52.8 MPa in the intact group, 56.3 MPa in the 1 mm group, 67.9 MPa in the 1.5 mm group, 62.1 MPa in the 2 mm group, 71.8 MPa in the 2.5 mm group, 70.3 MPa in the 3 mm group, 52.7 MPa in the 4 mm group and 55 MPa in the 5 mm group. The 4 mm and 5 mm groups showed similar stress results to the intact group (Fig. 4). Von Mises stress was markedly lower than the yield stress of the subchondral bone (135 MPa) in all groups [16].

### Analysis of safety factor in relation to distance between holes in simplified imaginary model

**Fig. 5:**
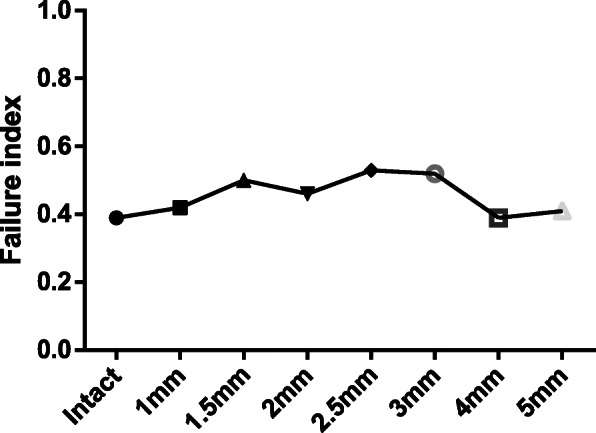
Safety factors in the 3D imaginary model

Because the modulus of elasticity and tensile strength of the subchondral plate varies, it is important to examine the safety factor. A safety factor (allowable stress/actual stress) of less than 1 is considered safe. The safety factor was 0.39 in the intact group, 0.42 in the 1 mm group, 0.50 in the 1.5 mm group, 0.46 in the 2 mm group, 0.53 in the 2.5 mm group, 0.52 in the 3 mm group, 0.41 in the 4 mm group and 0.39 in the 5 mm group (Fig. 5). Based on a mean safety factor of 0.41, it was confirmed that all distances were safe to a load of 147 N.

## Discussion

Microfracture is a minimally invasive, cost-effective, and relatively easy technique that induces knee cartilage repair by promoting an inflow of bone marrow stem cells to the cartilage defect site. Because of its advantages of low morbidity and little impact on future treatments, it has long been used as the preferential procedure for treating chondral injuries [17]. In a study that followed-up on patients who underwent microfracture for 3 years, 75% of the patients experienced pain relief [18]. Another study reported that microfracture led to clear improvements of various knee joint evaluation scores [19]. Activity-wise, a previous study reported that 86% of patients who underwent microfracture for a traumatic osteochondral lesion were able to resume their pre-trauma sports activities [6].

Despite its advantages, very few biomechanical studies on the microfracture technique have been performed. Although the current clinical guideline recommends a 3–4-mm distance between holes during microfracture [6, 7], there is no evidence supporting that this is the most mechanically stable method. Chen et al. argues that making holes in the subchondral bone would lead to changes in the subchondral bone structure, which would alter the biomechanics of the knee and ultimately influence cartilage repair, but this argument is not supported by mechanical evidence [20]. Recently, Shayan et al. conducted a finite element analysis to examine the changes of biomechanical properties after abrasion arthroplasty and microfracture, but they failed to analyse the biomechanical properties of subchondral bone after the microfracture technique [9]. In addition, it was difficult to accurately measure the material properties of the repaired cartilage, subchondral bone and trabecular bone, and they only examined changes in 2D cross sectional images and not in 3D overall structures.

Microfracture is a surgical technique that makes a cellular passage between the lesion and bone marrow to obtain cells required for chondral regeneration from the bone marrow. Therefore, it is speculated that making larger or more cellular passages would lead to improved chondral repair, as more bone marrow cells can be recruited to the lesion area, a notion supported by a recent study [4, 8, 21]. However, there have been no biomechanical studies investigating the relationship between subchondral bone stability and the microfracture holes made. In the current study, we set the distance between holes made during microfracture as the main variable to analyse the structural stability of the subchondral bone plate after microfracture using a finite element analysis. Because the number of holes was be altered depending on the distance between holes, we did not set it as one of the main variables for analysis.

We attempted to use a new modelling method that can replace the 3D computer aided design (CAD) modelling method used in previous studies. The image data used in previous studies to establish 3D models were low in resolution, so the resulting model was quite different from the actual shape of the subchondral bone. To improve the quality of the model to be used for the finite element analysis, we took micro-CT images of actual bone cartilage tissues removed during TKA and used the measurements for the subchondral bone measurements. Furthermore, we have devised a more anatomically and clinically reliable 3D imaginary model by simplifying the structure, making the femur side round while making the contacting tibia side flat. With this method, we were able to shorten the modelling time, model an object with a relatively complex structure and analyse the displacement within the structure, as well as the size and distribution of stress [22–25]. Hence, we examined the size of stress resulting from a load in relation to the distance between the holes in microfractures by comparing the peak Von Mises stress values as previously mentioned.

In this study, the peak von Mises values differed slightly in relation to the distance between holes, but the peak von Mises was considerably lower than the subchondral bone plate yield stress (135 MPa) [16]. Moreover, von Mises stress of the subchondral bone plate hardly increased at all, even when the distance between holes was set to 1 mm, and safety factors were all below 1 as well. These results suggest that the structural stability of the subchondral bone and trabecular bone is maintained irrespective of the distance between holes or the number of holes within the load used in this study. Based on these findings, it could be stated that the number of holes or distance between holes is not an important risk factor within the range of biomechanical load if the structures of the surrounding subchondral bone and trabecular bone are preserved well when making microfractures.

A limitation of this study was that the microfracture model was not made using conventional microfracture tools. The conventional microfracture awl tends to crush bone that may block microfracture holes placed close to each other [7] [25]. We used a previously published novel microfracture tool that results in a hollow trabecular hole that picks out bone rather than crushing bone, allowing for patent microfracture holes made 1–2 mm apart without compromising subchondral bone architecture [21]. Conventional microfracture tools may further compromise subchondral bone architecture in closely placed holes. Another limitation of this study was that the effects of the meniscus are ignored. Important functions of the meniscus include weight delivery, dispersion of external forces, protection of the articular cartilage, maintenance of articular stability and lubrication. Because the meniscus delivers 40–60% of the force load when standing, one major limitation of this study is that the meniscus was neglected. However, according to Krishnagoud Manda’s finite element analysis-based simulation model for treatment of a chondral defect by implanting a metal, removing the meniscus would have limited effects on the study’s findings. Further, we used a more extreme load in the present study, which would render the findings more reliable. The effects of neglecting meniscus on the outcomes seem trivial in this study [26].

This study aimed to analyse the structural stability of the subchondral bone in relation to the distance between the holes made in the microfracture technique. The results showed that the distance between holes, within the range used in this study, did not affect the structural stability. Based on this finding, it may be beneficial to make holes at shorter distances apart during microfracture to promote an influx of more MSCs from the bone marrow for better cartilage regeneration. However, this study performed a computer simulation with an imaginary loading condition and simplified imaginary model, and it is unknown whether the same results would be obtained in the anatomical joint conditions in clinical practice. We speculate that the actual clinical findings would be similar, as the subchondral bone area would be regenerated and become firm with the influx of MSCs from the bone marrow within 1–2 weeks of microfracture, which would disperse stress better. However, additional studies are needed to substantiate this.

## Conclusion

Although this study was limited in that it assumed that there was no surrounding subchondral bone tissue and sponge bone damage when making micro-perforations with an awl during the microfracture procedure, the findings of this study suggest that altering the distance between holes has little impact on the structural stability of the subchondral bone plate provided there are no problems that may affect the mechanical stability of surrounding tissues when making the holes, as assumed in this study. These results suggest that preserving the structure of surrounding tissues by improving the method of making microfractures would be an important topic for future mechanical research.

## Data Availability

All data generated or analysed during this study are included in this published article.

## Abbreviations

3D: Three-dimensional
FEM: Finite element model
MSC: Mesenchymal stem cell
TKA: Total knee arthroplasty
CT: Computed tomography
CAD: Computer aided design
